# Stat1 activation attenuates IL-6 induced Stat3 activity but does not alter apoptosis sensitivity in multiple myeloma

**DOI:** 10.1186/1471-2407-12-318

**Published:** 2012-07-28

**Authors:** Lina Y Dimberg, Anna Dimberg, Karolina Ivarsson, Mårten Fryknäs, Linda Rickardson, Gerard Tobin, Simon Ekman, Rolf Larsson, Urban Gullberg, Kenneth Nilsson, Fredrik Öberg, Helena Jernberg Wiklund

**Affiliations:** 1 Department of Immunology, Genetics and Pathology, Rudbeck Laboratory, Uppsala University, Uppsala, S- 751 85, Sweden; 2 Department of Medical Sciences, Uppsala University, Uppsala, Sweden; 3 Department of Laboratory Medicine, Lund University, Lund, Sweden

**Keywords:** Hematopoetic malignancies, Multiple myeloma, Apoptosis, IFN, Stat1, Stat3, Drug sensitivity

## Abstract

**Background:**

Multiple myeloma (MM) is at present an incurable malignancy, characterized by apoptosis-resistant tumor cells. Interferon (IFN) treatment sensitizes MM cells to Fas-induced apoptosis and is associated with an increased activation of Signal transducer and activator of transcription (Stat)1. The role of Stat1 in MM has not been elucidated, but Stat1 has in several studies been ascribed a pro-apoptotic role. Conversely, IL-6 induction of Stat3 is known to confer resistance to apoptosis in MM.

**Methods:**

To delineate the role of Stat1 in IFN mediated sensitization to apoptosis, sub-lines of the U-266-1970 MM cell line with a stable expression of the active mutant Stat1C were utilized. The influence of Stat1C constitutive transcriptional activation on endogenous Stat3 expression and activation, and the expression of apoptosis-related genes were analyzed. To determine whether Stat1 alone would be an important determinant in sensitizing MM cells to apoptosis, the U-266-1970-Stat1C cell line and control cells were exposed to high throughput compound screening (HTS).

**Results:**

To explore the role of Stat1 in IFN mediated apoptosis sensitization of MM, we established sublines of the MM cell line U-266-1970 constitutively expressing the active mutant Stat1C. We found that constitutive nuclear localization and transcriptional activity of Stat1 was associated with an attenuation of IL-6-induced Stat3 activation and up-regulation of mRNA for the pro-apoptotic Bcl-2 protein family genes Harakiri, the short form of Mcl-1 and Noxa. However, Stat1 activation alone was not sufficient to sensitize cells to Fas-induced apoptosis. In a screening of > 3000 compounds including bortezomib, dexamethasone, etoposide, suberoylanilide hydroxamic acid (SAHA), geldanamycin (17-AAG), doxorubicin and thalidomide, we found that the drug response and IC50 in cells constitutively expressing active Stat1 was mainly unaltered.

**Conclusion:**

We conclude that Stat1 alters IL-6 induced Stat3 activity and the expression of pro-apoptotic genes. However, this shift alone is not sufficient to alter apoptosis sensitivity in MM cells, suggesting that Stat1 independent pathways are operative in IFN mediated apoptosis sensitization.

## Background

Multiple myeloma (MM) is a malignancy characterized by an accumulation of plasma cells/ plasma blasts in the bone marrow. Common complications of MM include anemia, renal dysfunction, and bone destruction. Conventional drugs such as alkylating agents and corticosteroids, autologous and allogeneic bone marrow transplantation, bisphophanates, and novel drugs such as thalidomide, bortezomib, and lenalidomide can improve the quality of life and extend patient survival [1]. However, resistance to therapy often develops, making MM uniformly fatal.

A biological or mechanistic approach to combat drug resistance is to delineate the specific factors that are important in the regulation of growth, apoptosis and survival in MM. Thereby, putative critical drug targets for increased drug efficacy and circumvention of apoptosis resistance can be defined [2]. Ideally, therapy could be individualized based on the expression profile of the malignant plasma cells of the individual patient.

The aim of this study was to evaluate the importance of signal transducer and activator of transcription (Stat) 1 in influencing apoptosis and drug resistance in MM. Stat1 belongs to a family of transcription factors that are associated with regulation of growth and survival in hematopoietic cells. Upon cytokine stimulation, these proteins become phosphorylated by kinases, such as janus activated kinases (JAKs), associate with the cytoplasmic part of the cytokine receptor, form homo- or heterodimers and, finally, translocate to the nucleus where they bind to specific DNA elements and directly regulate transcription [3,4].

The Stat proteins Stat1 and Stat3 are constitutively activated in several types of tumors [5] including MM [6,7]. Stat3 is predominantly activated by the MM growth and survival factor IL-6. Stat1 is mainly activated by interferons, but can also to some extent be activated by IL-6 [8]. Stat3 has been defined as an oncogene in light of its ability to mediate cellular transformation and block apoptosis [9]. Some downstream targets of Stat3 that have been suggested to mediate these effects are anti-apoptotic Bcl-2-proteins and cell cycle regulators i.e. Bcl-XL, Mcl-1, c-Myc, and Cyclins D1/D2 [10]. In MM, the survival promoting effect of Stat3 has been ascribed to an up-regulated expression of the anti-apoptotic Bcl-2 family member Bcl-XL [6]. In contrast to Stat3, Stat1 has been proposed to promote apoptosis rather than survival [11-15]. It has been suggested that the relative abundance of the Stat1 and Stat3 proteins may influence the activity of each other. Supporting this hypothesis, Stat3 expression and activation is enhanced in Stat1 null cells [16] and, conversely, Stat1 expression and activation is enhanced in Stat3 null cells [17]. In ischemia / reperfusion mediated apoptosis, Stat1 was shown to promote apoptosis by down-regulating the Bcl-2 and Bcl-XL genes, whereas Stat3 counteracted this effect by up-regulating these genes [18]. Several inhibitors of Stat3 signaling have been developed (reviewed in [19]) for anti-cancer therapy, including a hairpin decoy oligonucleotide. This inhibitor was designed to block Stat3 but not Stat1 function, thereby presumably avoiding the tumor antagonist effects of Stat1 [20].

We have previously reported that MM cells that are highly resistant to Fas-induced apoptosis can be re-sensitized by pretreatment with IFN-γ [21]. One underlying cause for this effect was IFN-γ-induced up-regulation of the Fas receptor (CD95). Interestingly, we found that in addition to inducing and activating Stat1, IFN-γ also deactivated Stat3 [22]. In light of this observation, we hypothesized that one mechanism of IFN-γ mediated sensitization to apoptosis was deactivation of anti-apoptotic Stat3, possibly as a consequence of increased transcriptional activation and expression of Stat1.

To delineate the role of Stat1 in IFN mediated sensitization to apoptosis induced by Fas and therapeutically relevant drugs, we established sub-lines of the IL-6 dependent MM cell line U-266-1970 with a stable over-expression of wild type Stat1 as well as its active mutant Stat1C [23]. The Stat1C was utilized to obtain Stat1 homodimers and transcriptional activation also in the absence of IFN-γ induced tyrosine phosphorylation. To determine whether Stat1 alone would be an important determinant in sensitizing MM cells to apoptosis, the U-266-1970-Stat1C cell line and control cells were exposed to high throughput compound screening (HTS). In addition, we explored the influence of Stat1C constitutive transcriptional activation on endogenous Stat3 expression and activation, and the expression of apoptosis-related genes.

We report here that constitutive transcriptional activation of Stat1 in the U-266-1970 MM cell was indeed associated with an attenuation of Stat3 activation and a differential expression of several genes involved in apoptosis. However, Fas-induced apoptosis was not augmented in the U-266-1970-Stat1C cells, suggesting that increased activation of Stat1 transcription alone does not confer increased sensitization to Fas-induced apoptosis in MM. In a high through-put screening (HTS) of more than 3000 drugs, including bortezomib, dexamethasone, etoposide, suberoylanilide hydroxamic acid (SAHA), geldanamycin (17-AAG), doxorubicin and thalidomide, used in conventional and experimental MM therapy, as well as compounds from two annotated drug libraries, we found that IC50 was not reduced in U-266-1970-Stat1C. In fact, for two related agents, gitoxin and gitoxigenin, Stat1C conferred resistance rather than increased sensitivity. We conclude that shifting the balance from Stat3 to activated Stat1 in MM has an impact on transcriptional activation of genes i.e. Mcl-1, Noxa and Harakiri, but that this shift alone is not sufficient to alter sensitivity to IFN mediated apoptosis sensitivity or general drug response in MM cells.

## Methods

### Cell culture conditions

The IL-6 dependent MM cell line U-266-1970 [24] was maintained at 37°C in 5% CO_2_ in RPMI 1640 medium (Sigma Biosciences, St Louis, MO) supplemented with 10% heat inactivated fetal bovine serum (FBS; GIBCO, Grand Island, NY), 2 mM glutamine (Sigma), antibiotics (penicillin 100 U/mL and streptomycin 50 μg/mL) (Sigma), and 20 U/mL IL-6 (R&D Systems Europe Ltd, Abington, UK). For the stably transfected cells, the medium was also supplemented with G418 (Gibco). The cells were stimulated with IFN-γ, (1000 U/mL, Bender MedSystems GnbH, Vienna, Austria) as indicated. Unless stated otherwise, IL-6 was always present in the experiments. The U3A cell line, a Stat1-deficient mutant of the human fibroblast cell line 2ftgh cell line [25], was a kind gift from Dr. G.R. Stark. This cell line was maintained in Dulbeccos Modified Eagle´s Medium supplemented with 10% heat inactivated fetal bovine serum (FBS; GIBCO, Grand Island, NY), 2 mM glutamine (Sigma), antibiotics (penicillin 100 U/mL and streptomycin 50 μg/mL) (Sigma). All experiments were performed on exponentially growing cells.

### Plasmids for transient and stable Stat1transfections

The wtStat1pcIneo plasmid has been described previously [26]. The empty pcIneo vector (Promega) was used as a negative control. The FLAG-tagged STAT1C construct, consisting of a mutated form of Stat1 with Ala-656 and Asn-658 substituted for cysteine, a FLAG-tag, and a gene for neomycin resistance, was a kind gift from Dr. Sironi and Dr. Ouchi [23]. The pcDNA3.1.EGFP vector expressing green fluorescent protein was used as a marker for transient transfection (Biosciences).

### Transfections

To obtain cells with a stable expression of wtStat1 and Stat1C in U-266-1970 cells, the cells were nucleofected using the Amaxa Nucleofector™ apparatus (Amaxa, Cologne, Germany) according to the instructions of the manufacturer. Briefly, 2 x 10^6^ U-266-1970 cells were grown in cell medium without antibiotics for 24 hours and were then washed once in cold phosphate-buffered saline, and resuspended in 100 μl of electroporation buffer R with 4 μg of wtStat1pcIneo, empty pcIneo vector, or Stat1C vector as indicated. The cell suspension was the transferred to a 2.0 mm electroporation cuvette, and nucleofected with an Amaxa Nucleofector™ apparatus using nucleofection program U-05. 24 hours post-transfection IL-6 (20 U/ml) and G418 (1000 μg/ml) was added to the medium to select for stably transfected cells. After 6–10 weeks G418-resistant cells were maintained in cell medium with IL-6 (20 u/ml) and G418 (500 μg/ml).

For transient transfections of adherent U3A cells and 2ftgh, the calcium phosphate transfection method was used. Briefly, 6 x 10^6^ cells were seeded in each 10 cm plate the day before transfection. Chloroquine disphosphate was added 30 minutes before transfection to the cell culture plates. DNA (10 μg) was mixed with 0.25 M CaCl_2_. The mixture was then added to an equal volume of 2x HEPES-buffered saline, incubated for 20 minutes and finally added to the cells. The medium was changed and IFN-γ was added 6 hour post-transfection. The cells were harvested after 24 hours of IFN-γ stimulation.

### PCR

For identification of stably transfected cells expressing pcIneo and wtStat1 pcIneo, PCR was performed with forward primer 5’-AAG GCT AGA GTA CTT AAT ACG-3’ and reverse primer: 5’-ATT AAC CCT CAC TAA AGG GA-3’ (program:: 95° 7 min, (95° 25 s, 55° 25 s, 72° 30 s)x40, 72° 2 min). The reactions were performed using the Platinum Taq polymerase (Invitrogen) according to the manufacturer’s protocol.

### Western blot

U-266-1970 cells were incubated with 20 U/mL IL-6 alone or in combination with 1000 U/mL IFN-γ for the indicated time periods followed by harvest. For whole cell protein extracts, cells were washed once in PBS and lysed in 1% NP40, 0.1 M Tris–HCl, 0.15 M NaCl, 5 mM EDTA with protease inhibitors (1 mM ZnCl_2_, 50 mM Na_2_MoO_4_, 10 mM NaF, 0.1 mM NaVO_3_, 1 mM PMSF, 1 mM DTT, 1 x complete, EDTA-free (Roche, Mannheim, Germany) for 20 minutes at 4°C and centrifuged for 10 minutes at 4°C at 10000 X g to remove cellular debris. The supernatant was collected and the protein concentration was measured using the Bio-Rad protein assay (Bio-Rad Laboratories, Hercules, CA). After heating the samples at 70°C for 10 minutes the protein extracts were fractionated on NuPAGE Bis-Tris pre-cast gels (4-12%, 10%, 12%) using the Novex electrophoresis and blotting system (NOVEX, San Diego, CA). The membrane (Hybond-C extra, Amersham, United Kingdom) was blocked in 5% non-fat milk in TTBS (20 mM Tris–HCl (pH 7,5), 500 mM NaCl, and 5% Tween 20) at room temperature for 1 h, and then incubated at 4°C overnight with primary antibodies diluted in TTBS + 5% non-fat milk. The membrane was then washed 5x5 minutes in 1xTTBS, and incubated with secondary horseradish peroxidase (HRP)-linked antibodies (Amersham or, for detection of Stats and actin, DAKO, Glostrup, Denmark) diluted in TTBS + 5% non-fat milk for 1 hour at room temperature. After washing 5x5 minutes in 1xTTBS, protein detection by enhanced chemi-luminescence (ECL plus; Amersham, Bucks, U.K) was performed according to the manufacturer's protocol. Primary antibodies used where α-Bcl-x_L_ (H-62), α-Bcl-2 (C-21), α-actin (I-19), α-Stat1 (C-111), α-Stat3 (C-20), α-Mcl-1 (S-19), α-IRF-1 (C-20), α-Noxa (Santa Cruz Biotechnology, Santa Cruz, CA, USA), anti-Fas (UB2, Immunotech, Marseilles, France), anti-Histon H3 (ab1791) (abcam), anti-α-tubulin (A11126) (Invitrogen), rabbit polyclonal α-pStat1, rabbit polyclonal α-pStat3 (New England Biolabs, Beverly, MA, USA), α-FLIP (804-127-C100) (Alexis corporation, Läufelfingen, Switzerland), and α-FLAG (F-1804) (Sigma).

### Nuclear protein lysates

U-266-1970-Stat1C, and U-266-1970-pcIneo cells were incubated with IL-6 (20 U/mL) for 24 hours and were then stimulated with IL-6 alone or in combination with 1000 U/mL IFN-γ for the indicated time points. Nuclear and cytoplasmic protein lysates were prepared essentially as described by Andrews and Faller [27].

### Luciferase reporter assay

By using the Amaxa system as described, U-266-1970-pcIneo and U-266-1970-Stat1C cells were transiently transfected with 3 μg of the GBP-luc reporter vector (a kind gift from Dr. B. Lüscher, Hannover). The GBP-luc vector contains a luciferase reporter gene driven by a fragment of the human guanylate binding protein promoter containing Stat1-inducible ISRE and GAS elements [28]. To normalize the luciferase values due to differences in transfection efficiency, the cells were co-transfected with 1 μg of the vector hubactp/lacZ vector. This vector, which contains 4 kb of the human β-actin promoter linked to a lacZ reporter gene, was kindly provided by Dr. U. Lendahl [29]. Luciferase activity was measured in a luminometer (Lumat LB9501EG&G, Berthold, MA) using Luciferase Assay Reagent (Promega) according to manufacturer’s protocol. B-galactosidase activity was measured in the same luminometerusing Galacton and Emerald reagents (TROPIX; Bedford, USA) according to manufacturer’s protocol.

### Multiplex Ligation-Dependent Probe Amplification Assay (MLPA)

MLPA is a mRNA quantification assay based on probes, each of which consists of two unique oligonucleotides of specific lengths, one unlabeled and one fluorescently labeled, that anneal to adjacent sites on a specific target sequence. The annealed probes are ligated, allowing amplification of target sequences with specific lengths that can be quantified by capillary sequence analysis. All the probes used in the apoptosis gene probe set and detailed protocols have been described elsewhere [30,31]. U-266-1970-pcIneo and U-266-1970-Stat1C were incubated with 20 U/mL IL-6 (R&D Systems Europe Ltd, Abington, UK) alone or in combination with 1000 U/mL IFN-γ. The cells were harvested after 6 h and total RNA was prepared by Trizol extraction (Invitrogen) according to the manufacturer’s recommendation. Any contaminating DNA was removed using DNA-*free*™(Ambion). The MLPA was performed using the SALSA P011 Apoptosis mRNA MLPA kit (MRC-Holland , Amsterdam, The Netherlands) according to manufacturer’s recommendations. Briefly, 100 ng of RNA was first reverse transcribed using MMLV reverse transcriptase (Promega) together with the SALSA RT buffer and the SALSA RT primer mix. The resulting cDNA was hybridized to the SALSA probemix. Hybridized oligos were subjected to a ligation reaction, and ligation products were amplified by PCR using one labeled and one FAM-labeled primer. PCR products were analyzed on the ABI3700 capillary sequencer (Applied Biosystems) and peak areas representing gene expression was calculated using GeneScan software (ver3.7) for each gene. Data was normalized using the control gene ß-2 microglobulin, then all peak areas were summed to 100% and the relative peak area for each gene was calculated from this.

### Resazurin assay

Resazurin is the active compound of the commercially available compound Alamar Blue [32]. The resazurin assay can be used to quantify cell proliferation and cytotoxicity since the substance becomes increasingly fluorescent in the presence of metabolically active cells [33]. U-266-1970-pcIneo and U-266-1970-Stat1C were incubated with 20 U/mL IL-6 (R&D Systems Europe Ltd, Abington, UK) alone or in combination with 1000 U/mL IFN-γ in round bottomed 96-well plates. After the indicated time points, 10% Alamar Blue (resazurin) (Serotec Limited, Oxford, UK) was added to the wells, followed by incubation for 3 h at 37°C in a humidified 5% CO_2_ in-air atmosphere. The resazurin assay was also used to confirm the response to drugs identified in the high throughput FMCA screening. Analysis of fluorescence was performed by using a Wallac Victor Multilabel Counter (Wallac, Turku, Finland), where the resazurin was excited at 530 nm and the emitted light was measured at 590 nm. Mean was calculated from triplicate wells and subtracted from mean of blank wells resulting in ΔFluorescence. The relative number of viable cells was expressed as percentage of untreated cells and calculated as 100 x ΔFluorescence (treated cells)/ΔFluorescence (untreated cells).

### Flow cytometric analysis of apoptosis

U-266-1970 cells were incubated with 20 U/mL IL-6 alone or in combination with 1000 U/ml IFN-γ, and/or agonistic Fas-antibody CH-11 (100 ng/mL:, Immunotech, Marseilles, France) and/or isotype specific control IgM (X-0942, 100 ng/mL, DAKO A/S, Glostrup, Denmark). The percentage of Annexin V-positive/PI-negative apoptotic cells was measured on a FACScan flow cytometer and analyzed with CellQuest software (Becton Dickinson, San José, CA, USA), using the Annexin V-FITC kit (Calbiochem) or, when analyzing EGFP-transfected cells, with the Annexin V, Alexa Fluor 647 kit (Molecular Probes, Oregon, USA) according to the manufacturer’s protocol.

### Fluorometric Microculture Cytotoxicity Assay (FMCA)

The FMCA is an automated assay that measures fluorescence generated from hydrolysis of fluorescein diacetate to fluorescein by cells with intact plasma membranes [34]. In this assay, the sensitivity to drugs is determined by calculating a survival index (SI%), defined as the 100 x ΔFluorescence (treated cells)/ΔFluorescence (untreated cells), which is proportional to the number of living cells. Cell cultures were seeded in drug-prepared plates up in 384 well plates at a density of 10 000 cells/ well and treated as described elsewhere [35]. The drugs used in the assay were etoposide, SAHA, dexamethasone, doxorubicin, As203, bortezomib, irinotecan, amsacrine, parthenolide, fludarabine, IMD 0354, 17-AAG/geldanamycin, TBB, docetaxel, oncovin, and resveratrol. For each drug, five different concentrations were used. For each drug, IC_50_-values (inhibitory concentration 50%) were calculated by using non-linear regression and a standard sigmoidal dose–response model in the GraphPadPrism program (GraphPad Software, Inc. San Diego, CA, USA). The FMCA assay was also used to screen the Library of pharmacologically active compounds (LOPAC1280) (Sigma-Aldrich, St. Louis, Mo.) consisting of 1266 drugs from 56 pharmacological classes and the Spectrum Collection (MicroSource Discovery Inc., Groton, Conn.) consisting of 2000 drug components, natural products and bioactive components. All the LOPAC drugs were screened at a concentration of 10 μM. An Accord HTS database (Accelrys Inc, San Diego, CA, USA) and Small Laboratory Information and Management System (SLIMS: Kelley, Lunn, Stockwell 2004) were used for screening data management and analysis. Raw data files were loaded into the SLIMS software which calculates percent inhibition according to the formula: Percent inhibition = 100 x (test well-blank/control-blank)-1.

## Results

### Establishment of U-266-1970 cells sublines with a stable expression of Stat1C

**Figure 1:**
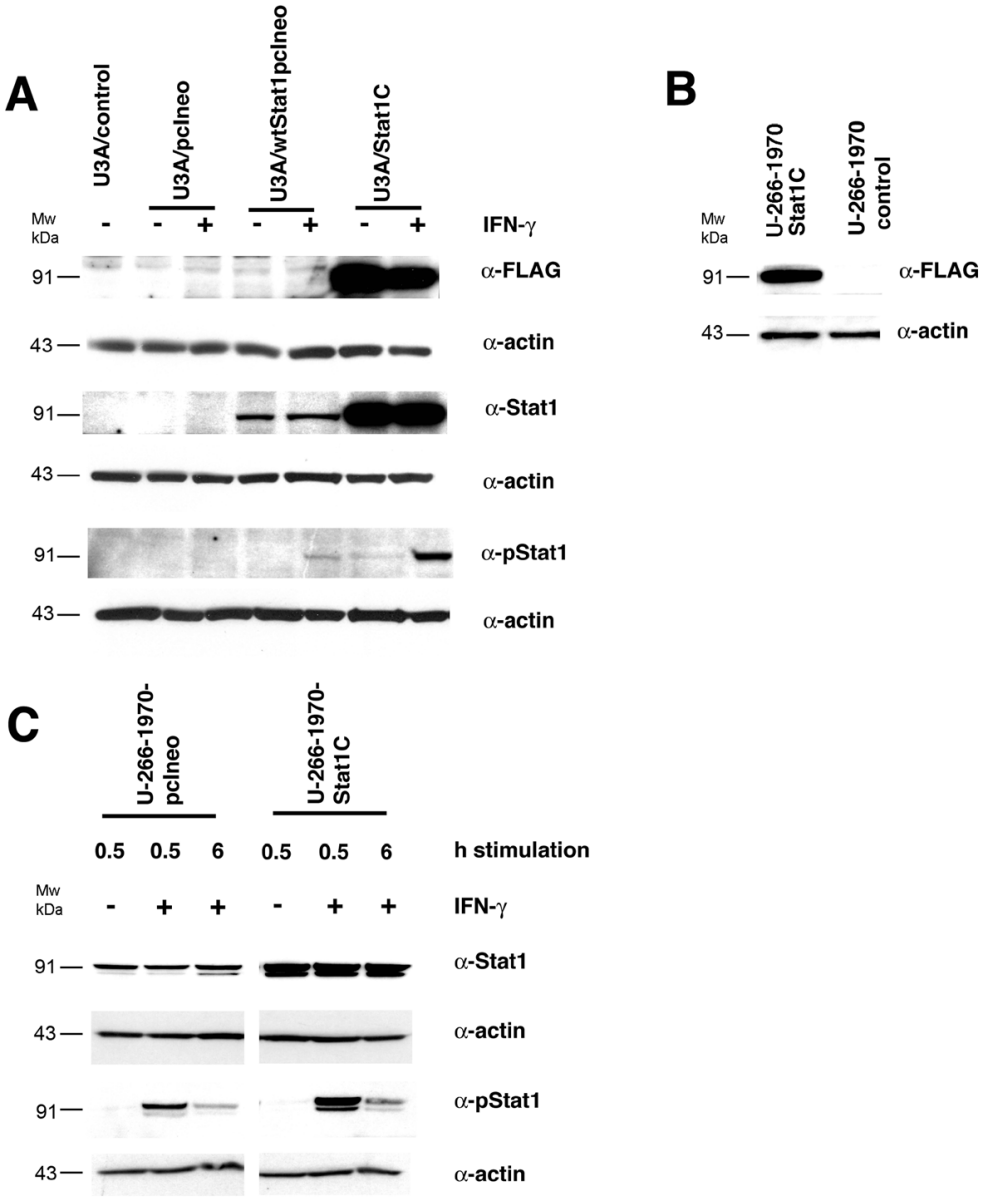
** Protein expression of Stat1, P-Stat1 and FLAG in transfected cells.** ( **A**) U3A cells were transiently transfected with the pcIneo vector, the wtStat1pcIneo vector and the Stat1C vector as indicated. The cells were left untreated or were treated with IFN-γ (1000 U/mL) for 24 hours. The cells were lysed and Western blot analysis was performed using the indicated antibodies. ( **B**) and ( **C**) Stably transfected U-266-1970 cells were left untreated or were treated with IFN-γ (1000 U/mL) for the indicated times. Protein lysates were prepared and Western blot analysis was performed using the indicated antibodies.

With the aim of evaluating the role of constitutive transcriptional activity and expression of Stat1, we established sublines of U-266-1970 cells expressing wild type Stat1 or the active mutant Stat1C [23], where the Ala-656 and Asn-658 in the SH2-domain have been exchanged for cysteine to promote spontaneous dimerization and constitutive activation of the Stat1C protein. Besides the activating mutation, the Stat1C vector also offers the advantage of a FLAG-tag in its C-terminus, enabling discrimination between the transfected gene and endogenous Stat1 protein. Transfection of Stat1-deficient U3A-cells with vectors expressing wtStat or Stat1C restored Stat1 expression (Figure 1A). Also, IFN-γ treatment induced Stat1 phosphorylation in the Stat1C mutant. U-266-1970 MM cells were stably transfected with wtStat1pcineo, Stat1C, and empty vector pcIneo, respectively, and G418-resistant cells were collected after 6–10 weeks. The expression of wtStat1pcineo and empty vector pcIneo was confirmed by PCR, using primers specific for the vector sequence (data not shown). The expression of the Stat1C vector FLAG-tag protein was confirmed by Western blot (Figure 1B).

To evaluate Stat protein expression and activation in the different sublines (designated U-266-1970-wtStat1pcIneo (data not shown), U-266-1970-Stat1C, and U-266-1970-pcIneo), we stimulated the cells with IFN-γ and harvested them at the indicated time points. Stat1C-transfected cells displayed a pronounced increase in Stat1 expression and a marked enhancement of IFN-γ induced tyrosine phosphorylation (Figure 1C). The U-266-1970-Stat1C subline was chosen for further analysis outlining the role of Stat1 on gene expression and apoptosis sensitization.

### Nuclear translocation of Stat1C and increased transcriptional activity in U-266-1970-Stat1C cells

**Figure 2:**
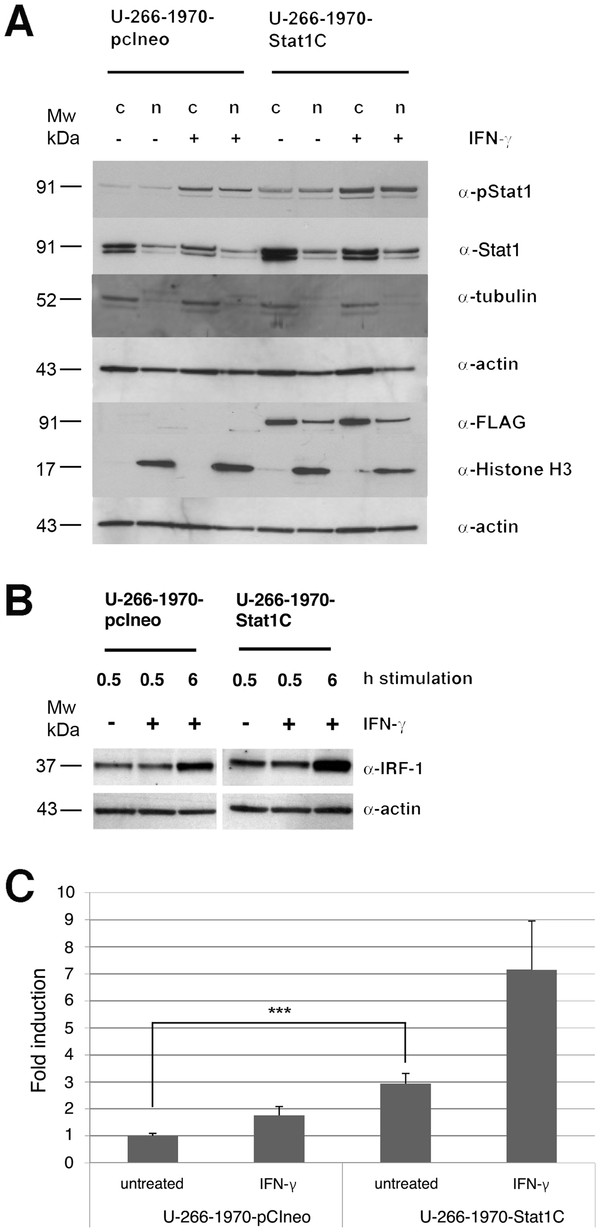
** Nuclear translocation and transcriptional activity of stably transfected cells.** Stably transfected U-266-1970-pcIneo cells and U-266-1970-Stat1C cells were left un-treated or were treated with 1000 U/mL IFN-γ for 0.5 hours. Nuclear and cytoplasmic protein extracts were prepared. ( **A**) Western blot analysis of the nuclear (n) and cytoplasmic (c) protein extracts were performed using the indicated antibodies. The proteins α-tubulin and α-histone H3 are markers for the cytoplasmic and nuclear fraction respectively. ( **B**) Stably transfected U-266-1970-pcIneo or U-266-1970-Stat1C cells were left untreated or were treated with IFN-γ (1000 U/mL) for the indicated times. Protein lysates were prepared and Western blot analysis was performed using the indicated antibodies. ( **C**) The luciferase reporter contruct GBP-luc was cotransfected with hubactp/lacZ vector into U-266-1970-pcIneo cells and U-266-1970-Stat1C cells as described. After 24 hours of treatment with IFN-γ (1000 U/mL), protein extracts were prepared and luciferase assays were performed. The graph shows fold induction of relative light units (RLU) as the mean of three independent experiments ± SD. Corrections have been made for varying transfection efficiency.

To confirm the constitutive transcriptional activity of the transfected Stat1C protein we evaluated the potential of Stat1C to translocate to the nucleus in untreated cells and in response to IFN-γ stimulation. U-266-1970-Stat1C and U-266-1970-pcIneo cells were treated with IFN-γ at the indicated times, the cells were harvested and nuclear and cytoplasmic protein lysates were prepared and compared to untreated control cells. As depicted in Figure 2A, in U-266-1970-Stat1C cells, the FLAG protein was present in both the cytoplasmic and the nuclear fraction of stimulated and un-stimulated cells. The results show that transfected Stat1C translocates to the nucleus also in the absence of IFN-γ-induced tyrosine phosphorylation, thereby suggesting that the protein has the potential to influence gene transcription constitutively. Upon IFN-γ stimulation, phosphorylated Stat1 was present in both the cytoplasmic and the nuclear fraction of both sublines.

To confirm that the nuclear presence of Stat1C in the U-266-1970-Stat1C sub-line was reflected by an increased Stat1-induced transcriptional activation, we analyzed the protein expression of the well-characterized IFN-inducible gene interferon regulatory factor 1 (IRF-1) in the sublines. Figure 2B shows that both the basal expression and the IFN-γ induced expression of IRF-1 were significantly increased in the Stat1C transfected subline (see also Additional file 1: Figure S1). Consistent with increased Stat1 activity we also observed a statistically significant (>2-fold) higher transcription from GBP-luc, a luciferase reporter containing Stat1-inducible ISRE and GAS elements, in Stat1C expressing U-266-1970 cells as compared to untreated control cells (Figure 2C). As expected, the increase in transcriptional activity was further enhanced by the treatment of IFN-γ in both U-266-1970-Stat1C and U-266-1970-pcIneo cells.

### Regulation of apoptosis-related genes in U-266-1970-Stat1C cells

**Figure 3:**
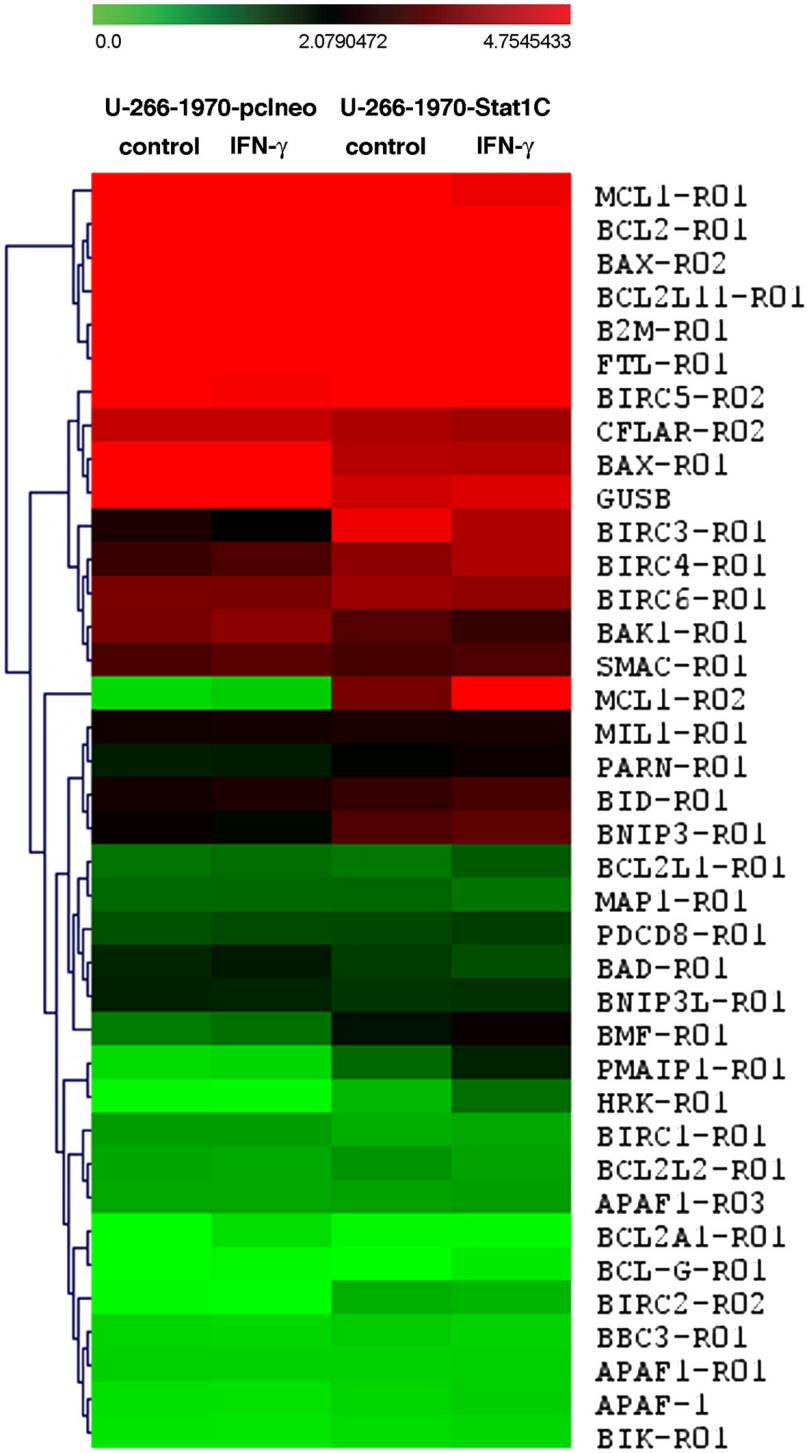
** MLPA Apoptosis gene expression profile of U-266-1970-pcIneo cells and U-266-1970-Stat1C cells.** The cells were left untreated or were treated with IFN-γ for 6 hours as indicated, RNA lysates were prepared and subjected to MLPA analysis as described. Average relative gene expression from three independent experiments performed in duplicates, are displayed (one pcIneo-sample and one Stat1C-sample were lost). The relative mRNA gene expression is color coded from green (no expression) to red (highest expression) (see bar at the top). The MLPA probes are indicated on the right. All genes that differed more than 2x between empty-vector-transfected and Stat1C-expressing sub-lines are summarized in Table 1.

**Figure 4:**
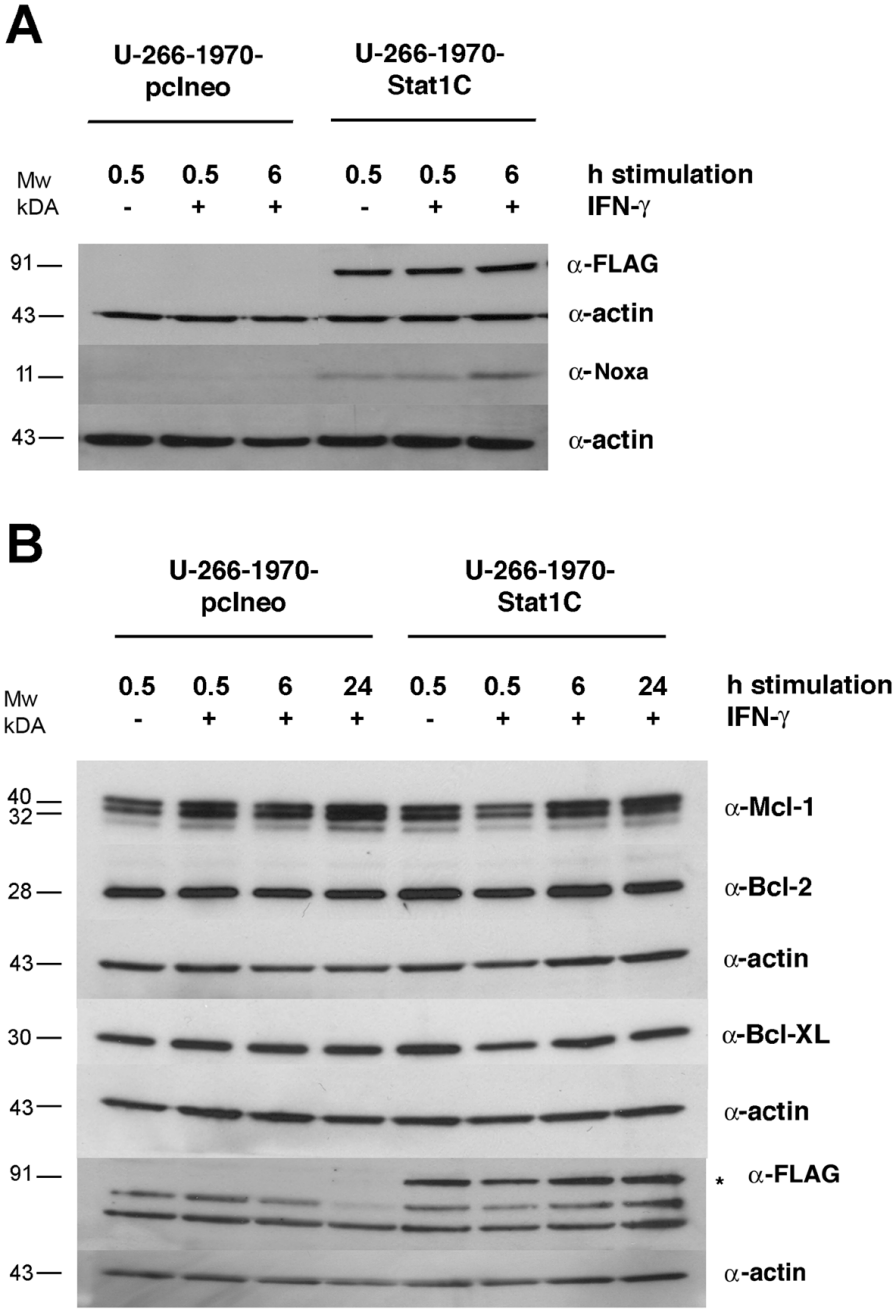
**Protein expression of Mcl-1, Bcl-XL, Bcl-2 and Noxa in U-266-1970-pcIneo cells and U-266-1970-Stat1C cells. **( **A**) and ( **B**) U-266-1970-pcIneo cells and U-266-1970-Stat1C cells were left untreated or were treated with IFN-γ (1000 U/mL) for the indicated times. Protein lysates were prepared and Western blot analysis was performed using the indicated antibodies.

Next, we examined how the constitutively active Stat1 would influence the expression of apoptosis-related genes in MM. For this purpose, we used the SALSA P011 Apoptosis mRNA Multiplex Ligation-Dependent Probe Amplification Assay (MLPA) kit. This assay quantifies the relative mRNA expression of 39 different probes corresponding to apoptosis-related genes, including 21 different Bcl-2 family genes, 7 members of the IAP family, and other pro- and anti-apoptotic proteins such as Apaf-1, Smac/DIABLO, and Flip. Figure 3 illustrates the gene expression of un-stimulated and IFN-γ- stimulated U-266-1970-pcIneo cells and U-266-1970-Stat1C cells. We considered a difference in gene expression to be significant if quantified to be at least two-fold, in addition to being statistically significant as calculated by the Student’s *t*-test. Genes that differed significantly according to these criteria are summarized in Table 1. Notably, Stat1C expression was associated with increased mRNA levels of Harakiri, the short form of Mcl-1 and Noxa genes. Accordingly, Noxa protein expression was increased in the Stat1C cells as shown by Western blot analysis (Figure 4A). The protein expression of Harakiri, a small protein that is notoriously difficult to detect by Western blotting, could not be assayed (results not shown).

**Table 1:** Differentially expressed genes in U-266-1970-Stat1C cells versus U-266-1970-pcIneo cells, untreated or IFN-γ-treated

**MLPA probe**	**Corresponding gene**	**Category**	**Fold expression in Stat1C vs pcIneo**	**Fold expression in Stat1C/IFN-γ vs pcIneo/IFN-γ**
PMAIP1-RO1	Noxa	Bcl-2 family	4.4	5.6
		BH3-only		
		Pro-apoptotic		
HRK-R01	Harakiri	Bcl-2 family	9.1	28.5
		BH3-only		
		Pro-apoptotic		
MCL1-R02	Mcl-1 short	Bcl-2 family	11.2	13.4
		Bax-like		
		Pro-apoptotic		
BCLG-R01	Bcl-GS	Bcl-2 family	n.s.	2.8
		Bax-like		
		Pro-apoptotic		

(P < 0.005, > 2-fold difference).

The MLPA analysis did not reveal a regulation of the Bcl-2 and Bcl-XL genes at the transcriptional level. However, given the previously established link between these proteins, Stat activation and survival [6,18], we were interested in whether the level of Bcl-2 and Bcl-XL protein expression would differ between the U-266-Stat1C cell line and the vector transfected subline. In addition to these genes, the protein expression of Mcl-1 was assayed, the short form of Mcl-1 being the gene most consistently up-regulated at the mRNA level in the Stat1C subline. As shown in Figure 4B, the Bcl-2, Bcl-XL and Mcl-1 were, however, not significantly different at the protein expression level in the U-266-1970-Stat1C sub-line. IFN-γ stimulation did not have any major effect on the protein expression of any of the genes examined (see also Additional file 1: Figure S2).

### Stat1 activation attenuates IL-6 induced Stat3 activity in U-266-1970 cells

**Figure 5:**
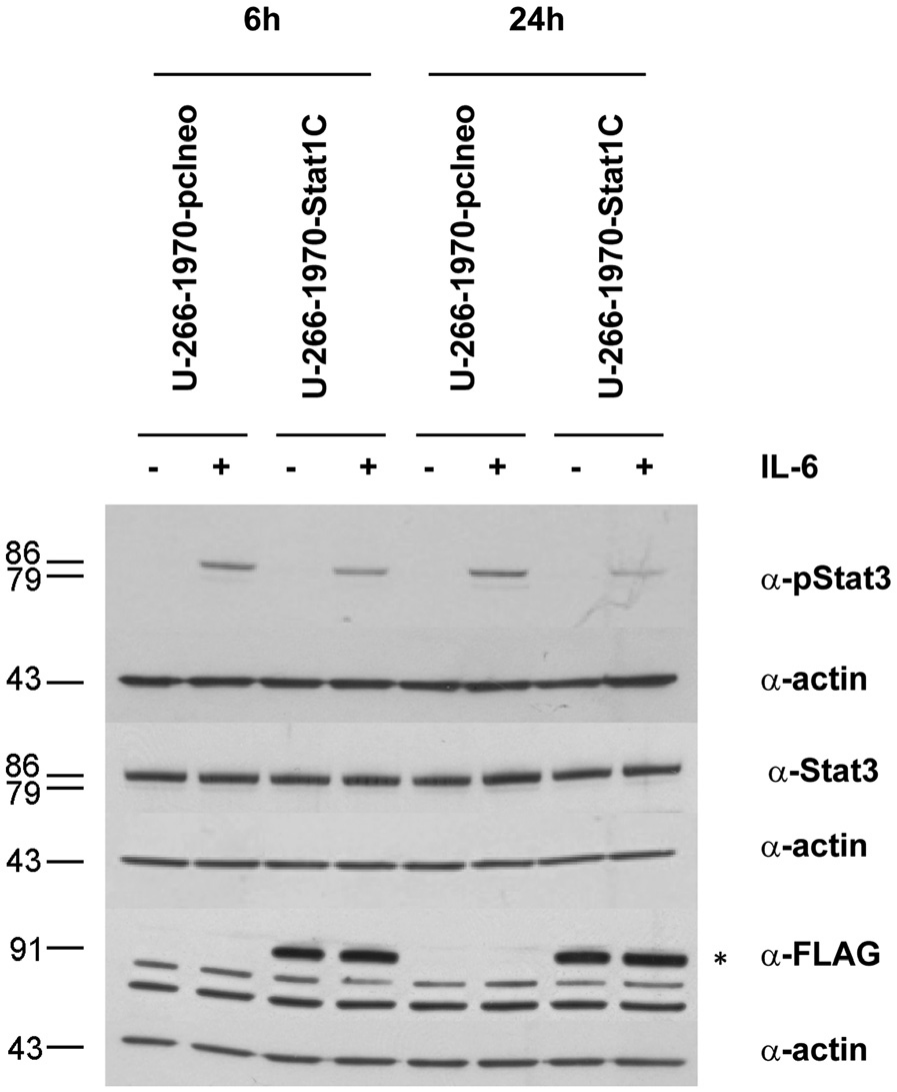
** Protein expression of Stat3 and pStat3 in U-266-1970-pcIneo cells and U-266-1970-Stat1C cells.** U-266-1970-pcIneo cells and U-266-1970-Stat1C cells were left untreated or were treated with IL-6 (20 U/mL) for the indicated times. The cells were IL-6-deprived overnight prior to stimulation and harvest. Protein lysates were prepared and Western blot analysis was performed using the indicated antibodies.

We have previously shown that in addition to activating Stat1, IFN-γ also deactivates Stat3 [22]. We hypothesized that Stat1 activation has a negative effect on Stat3 activation, counteracting the pro-survival properties of Stat3 and thereby augmenting apoptosis sensitivity. Figure 5 shows Western blots of untreated and IL-6 treated U-266-1970-pcineo cells and U-266-1970-Stat1C cells. Stat3 was examined after IL-6-induction in IL-6 deprived U-266-1970-pcIneo and U-266-1970-Stat1C cells at 6 and 24 hours. In support of our hypothesis, the level of phosphorylated Stat3 in response to IL-6-stimulation at 6 and 24 hours were attenuated in the U-266-1970-Stat1C subline as compared to U-266-1970-pcIneo cells. As depicted the levels of total Stat3 protein were unaltered in U-266-1970-Stat1C cells as compared to U-266-1970-pcIneo cells (see also Additional file 1: Figure S3).

### The effect of Stat1 on the sensitivity to Fas-induced apoptosis in MM cells

**Figure 6:**
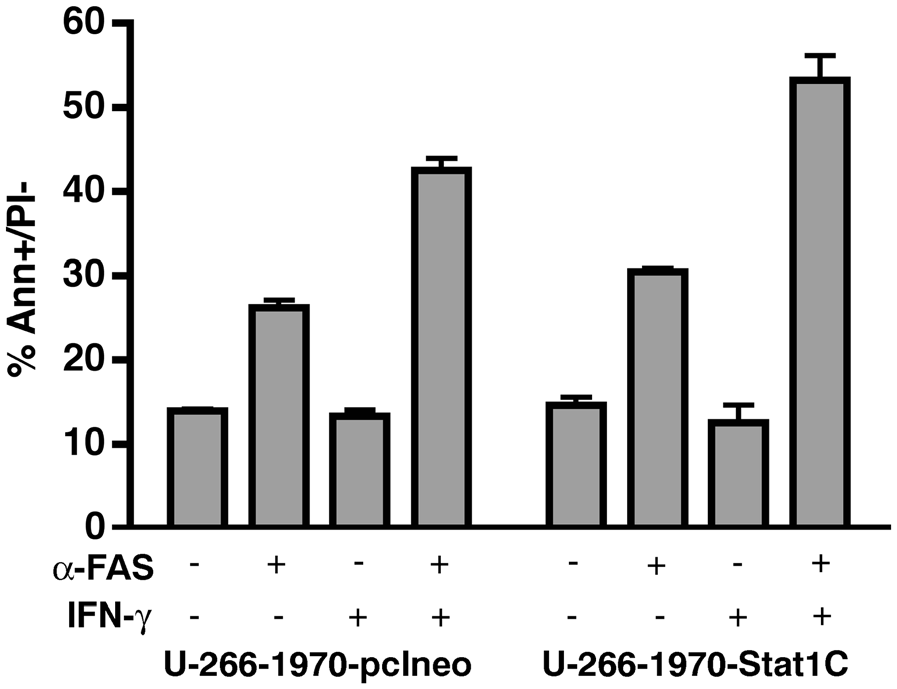
** Stat1C alone does not confer sensitivity to Fas-induced apoptosis.** U-266-1970 cells and U-266-1970-Stat1C cells were left untreated or were treated with IFN-γ (1000 U/ml) as indicated for 96 hours, followed by a 24 hour incubation with agonistic Fas-antibody CH-11 (FAS) as indicated. The percentage of Annexin V-positive/PI-negative apoptotic cells was evaluated by flow cytometry as described in Materials and Methods. The graph shows the percentage of AnnexinV positive, PI-negative cells, i.e. early apoptotic cells, as a mean of three experiments ± SD.

Our previous studies have shown that IFN-γ sensitizes myeloma cells for apoptosis by the death receptor Fas [21]. To evaluate whether the constitutive activation of Stat1 would render the cells more sensitive to Fas-induced apoptosis, we stimulated the Stat1C expressing subline and the U-266-1970-pcIneo cells with the Fas receptor agonistic antibody, with or without prior IFN-γ -stimulation. The proportion of apoptotic, Annexin V positive and propidium iodide negative cells were determined by flow cytometry as described. As depicted in Figure 6, stimulation with IFN-γ prior to Fas-exposure increased the percentage of apoptotic cells in the U-266-1970-pcIneo control cells as compared to cells stimulated by Fas only. The sensitizing effect of IFN-γ was sustained in the U-266-1970-Stat1C cells. Importantly, transcriptionally active Stat1 alone did not significantly enhance the apoptotic response to Fas in the absence of IFN. Confirming our previous findings, stimulation with IFN-γ prior to Fas-exposure increased expression of Fas receptor in both sublines [22] (See Additional file 1: Figure S4). These data suggests that activation of Stat1 per se is not sufficient to significantly sensitize U-266-1970 cells to Fas-induced apoptosis in the absence of IFN-γ stimulation, indicating that the effect of IFN-γ on Fas-induced apoptosis is not solely mediated by Stat1 activation.

### The effect of Stat1 on drug sensitivity in MM cells

**Figure 7:**
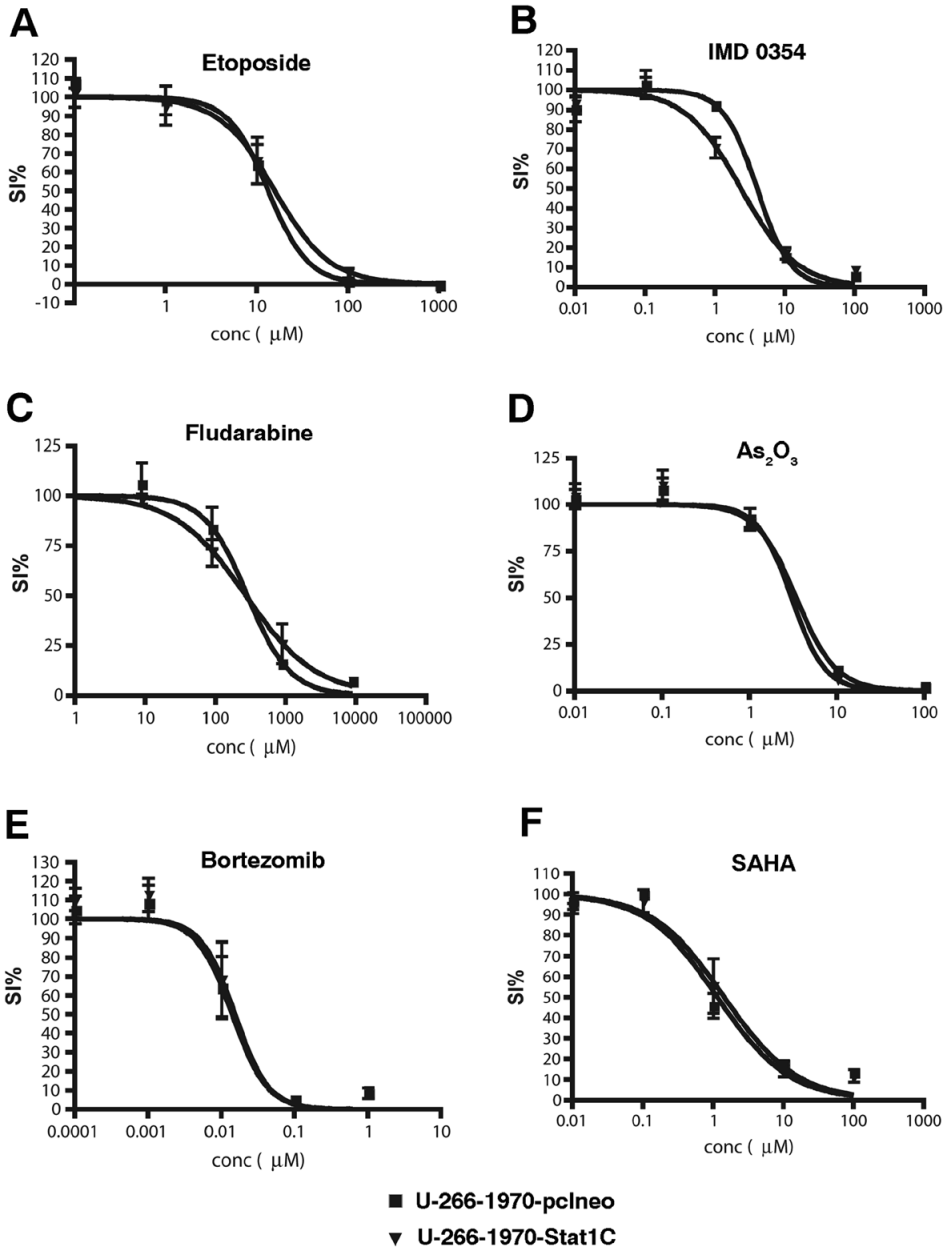
** Dose–response curves displaying the sensitivity of U-266-1970-pcIneo cells and U-266-1970-Stat1C cells to different drugs.** Using the FMCA assay as described, the response to indicated concentrations of ( **A**) Etoposide, ( **B**) IMD 0354, ( **C**) Fludarabine, ( **D**) As2O3, ( **E**) Bortezomib, ( **F**) SAHA, in U-266-1970-pcIneo cells and U-266-1970-Stat1C cells was monitored. Each data point represents the mean of three independent experiments ± SD.

To determine if transcriptionally active Stat1 affects the sensitivity to apoptosis in general, we selected 16 different drugs on the basis of MM therapeutic relevance, previously established connection to Stat1 or Stat3 activation, and/or novelty, and tested the survival index of each drug in a broad concentration range using the FMCA assay. The responses to six of these drugs (Etoposide, IMD 0354, Fludarabine, As_2_O_3_, Bortezomib, and SAHA) are graphed in Figure 7. There was no significant difference between the U-266-1970-Stat1C sub-line and the vector transfected control cells in the sensitivity to the drugs tested (Figure 7 and data not shown).

In an unbiased high throughput screening, we exposed the sublines to the 1266 drugs of the LOPAC library and to the 2000 pharmacologically active substances of the Spectrum Collection library. Only 18 of the drugs of the LOPAC induced a survival index lower than 50% at a concentration of 10 μM in any of the sub lines (data not shown), confirming the fact that myeloma cells are highly resistant to apoptosis. We arbitrarily considered 20% of difference in survival index between empty-vector transfected and Stat1-transfected sub-lines to be a potentially relevant difference. Several drugs induced a differential response in the cell lines using this criterium, but when repeating the experiments on a smaller scale using resazurin assay, only 2 drugs (gitoxin and gitoxigenin) could be confirmed as yielding statistically significant changes in apoptosis sensitivity in the two cell lines, the Stat1 expressing cells being more resistant than control cells (Additional file 1: Figure S5).

## Discussion

An important challenge in MM research lies in pinpointing the critical factors that regulate growth and survival so that potential therapeutic targets can be defined. Stat1 is constitutively activated in many MM patients [6], and has been described as a pro-apoptotic protein in a number of studies [12-14]. In contrast, the family member Stat3, has been identified as a pro-survival protein activated downstream the MM growth- and survival factor IL-6 [6,7,36,37] and as a promising drug target [19,20]. Both proteins can be regulated by IL-6 and by interferons, they can bind to the same DNA motifs, they are highly homologous to each other, and they can form heterodimers of Stat1/Stat3 that may have other specificities in gene regulation as compared to either homodimer alone [38]. It has also been suggested that Stat1 and Stat3 can oppose the effects of each other [16,39-41], for instance by exerting opposing effects on the promoters of pro- or antiapoptotic genes [18].

We have previously found that IFN-γ sensitizes MM cells for apoptosis induced by the death receptor Fas [21] by a mechanism involving up-regulation of the Fas receptor [22]. Intriguingly, we found that IFN-γ stimulation induces not only Stat1 activation but also Stat3 deactivation [22]. In the present study we show that Stat3 protein was decreased and phosphorylation of Stat3 was attenuated in an IL-6 dependent MM cell line, the U-266-1970 , expressing a constitutively active mutant of Stat1, the Stat1C. This observation is in line with other studies that have shown enhanced Stat3 expression and activation in Stat1 null cells [16] and, conversely, enhanced Stat1 expression and activation in Stat-3 null cells [17]. It is also consistent with the finding that IFN-α induced apoptosis of MM cells is associated with inhibition of Stat3 activity [42]. Since Stat1 and Stat3 are often expressed simultaneously in the same tumor cells, it has been suggested that one protein dominates over the other in the influence on survival, depending on e.g. the durability of activation of each protein [43]. The Stat1C protein would be expected to function qualitatively as endogenous Stat1 and becomes phosphorylated on Y701 and S727 by interferon-treatment [23] although it is constitutively present in the nucleus also in the absence of IFN treatment. In the present study we show that Stat1C- expressing cells with constitutive transcriptional activity were sensitized to Fas induced apoptosis by IFN-γ, but that apoptosis induced by the Fas receptor alone was not enhanced in Stat1C- expressing cells as compared to empty vector-transfected cells. This data is consistent with our previous findings [21,22], but suggests that Stat1 up-regulation is not a sole mediator of the apoptosis sensitizing effect of IFN-γ.

We found that the expression of Stat1C influenced the expression of the apoptosis-related gene Mcl-1 s, while the expression of Bcl-2 and Bcl-xL was not significantly altered in the Stat1C sub-line. Mcl-1 s has previously been characterized as important regulators of survival in MM and interesting therapeutic targets [44,45]. Harakiri, a pro-apoptotic member of the Bcl-2 family, was also up-regulated in the Stat1C-expressing sub-line. This protein interacts specifically with Bcl-2 and Bcl-XL, counteracting their anti-apoptotic effect [46]. The mRNA expression of the pro-apoptotic Bcl-2 protein Noxa was strongly augmented in U-266-1970-Stat1C. Interestingly, Noxa has previously been shown to be down-regulated in Stat1 (−/−) cells [47]. Its expression has been associated with, amongst others, p53-mediated apoptotic response [48].

Despite the up-regulation of the pro-apoptotic proteins, introduction of the Stat1C mutant in MM cells did not affect apoptosis in response to any of the 16 drugs selected on the basis of relevance for MM therapy, known Stat1 or Stat3 influence, or novelty. In the HTS of drugs from the LOPAC library and the Spectrum collection we did not observe a general Stat1-dependent difference in apoptosis-sensitivity. However, we could confirm that Stat1C-expressing MM cells were significantly more resistant in response to two similar compounds – gitoxin and gitoxigenin. A major role of Stat1 in promoting apoptosis have previously been challenged by studies showing increased Stat1 expression in cells with acquired resistance to chemotherapeutic drugs and radiation [49,50]. At present the mechanism(s) for these observations are unknown, but may involve other partners of Stat1 i.e. the ISGF3 (Stat1/Stat2/IRF9 complex) mediated signaling [49,50].

## Conclusions

We conclude that constitutive transcriptional activation of Stat1 alters IL-6 induced activation by Stat3. However, this shift alone is not sufficient to alter sensitivity to apoptosis induced by Fas receptor or sensitivity to therapeutic drugs in MM. This study suggests that also Stat1 independent pathways are operative in IFN mediated apoptosis sensitization and underlines the importance of understanding the details of MM survival pathways in order to exploit them as therapeutic targets.

## Competing interests

The authors declare that they have no competing interests.

## Authors’ contributions

LYD designed and performed the experiments, analyzed the data and wrote the paper, AD designed the experiments, analyzed the data and wrote the paper, KI performed the experiments, MF contributed analysis tools, LR contributed analysis tools, GT performed the experiments and analyzed the dat, SE analyzed the data, RL contributed analysis tools, UG contributed reagents, KN critically revised the paper, FÖ conceived the experiments, analyzed data and contributed reagents, HJW conceived and designed the experiments and supervised the project. All authors read and approved the final manuscript.

## Pre-publication history

The pre-publication history for this paper can be accessed here:

http://www.biomedcentral.com/1471-2407/12/318/prepub

## Supplementary Material

Additional file 1 ** Figure S1.** (A) U-266-1970-pCIneo and U-266-1970-Stat1C were induced by IFN-γ (1000U/mL) for the indicated times. Quantification of w.b. for independent biological replicates (n = 3). A representative experiment is depicted in Figure 2B. Expression of IRF-1 relative to Actin ± S.D. is shown. * p-value < 0.024. **Figure S2.** U-266-1970-pCIneo and U-266-1970-Stat1C were induced by IFN-γ (1000U/mL) for the indicated times. Quantification of w.b. for independent biological replicates (n = 3). A representative experiment is depicted in Figure 4B. Expression of MCL-1, BCL-2 and BCL-XL relative to Actin ± S.D. is shown. **Figure S3.** Quantification of the w.b. shown in Figure 5. (A) phospho-Stat3 expression in U-266-pCIneo and U-266-Stat1C treated with IL-6 (20U/mL) for 6 and 24 hours, and (B) showing total Stat3 expression for the same experiment. **Figure S4.** U-266-1970-pCIneo and U-266-1970-Stat1C were induced by IFN-γ (1000U/mL) for 96 h. Apoptosis was induced as shown in Figure 6. Expression of CD95/Fas analysed by Flow cytometry analysis (n = 3) is indicated as mean flourescense intensity (MFI). **Figure S5.** Response to Gitoxin and Gitoxigenin at 72 h in U-266-1970-pcIneo cells and U-266-1970-Stat1C cells using the Resazurin assay. Each data point represents the mean of three independent experiments ± SD. (DOC 445 kb) Click here for file
