# Supplementary material for: Stat1 activation attenuates IL-6 induced Stat3 activity but does not alter apoptosis sensitivity in multiple myeloma
Source: BMC Cancer. 2012 Jul 28;12:318. doi: 10.1186/1471-2407-12-318 (PMC3488543; doi:10.1186/1471-2407-12-318)
Supplement: Additional file 1 — Figure S1. (A) U-266-1970-pCIneo and U-266-1970-Stat1C were induced by IFN-γ (1000U/mL) for the indicated times. Quantification of w.b. for independent biological replicates (n = 3). A representative experiment is depicted in Figure 2B. Expression of IRF-1 relative to Actin ± S.D. is shown. * p-value < 0.024. Figure S2. U-266-1970-pCIneo and U-266-1970-Stat1C were induced by IFN-γ (1000U/mL) for the indicated times. Quantification of w.b. for independent biological replicates (n = 3). A representative experiment is depicted in Figure 4B. Expression of MCL-1, BCL-2 and BCL-XL relative to Actin ± S.D. is shown. Figure S3. Quantification of the w.b. shown in Figure 5. (A) phospho-Stat3 expression in U-266-pCIneo and U-266-Stat1C treated with IL-6 (20U/mL) for 6 and 24 hours, and (B) showing total Stat3 expression for the same experiment. Figure S4. U-266-1970-pCIneo and U-266-1970-Stat1C were induced by IFN-γ (1000U/mL) for 96 h. Apoptosis was induced as shown in Figure 6. Expression of CD95/Fas analysed by Flow cytometry analysis (n = 3) is indicated as mean flourescense intensity (MFI). Figure S5. Response to Gitoxin and Gitoxigenin at 72 h in U-266-1970-pcIneo cells and U-266-1970-Stat1C cells using the Resazurin assay. Each data point represents the mean of three independent experiments ± SD. (DOC 445 kb) [file 1471-2407-12-318-S1.doc]

**
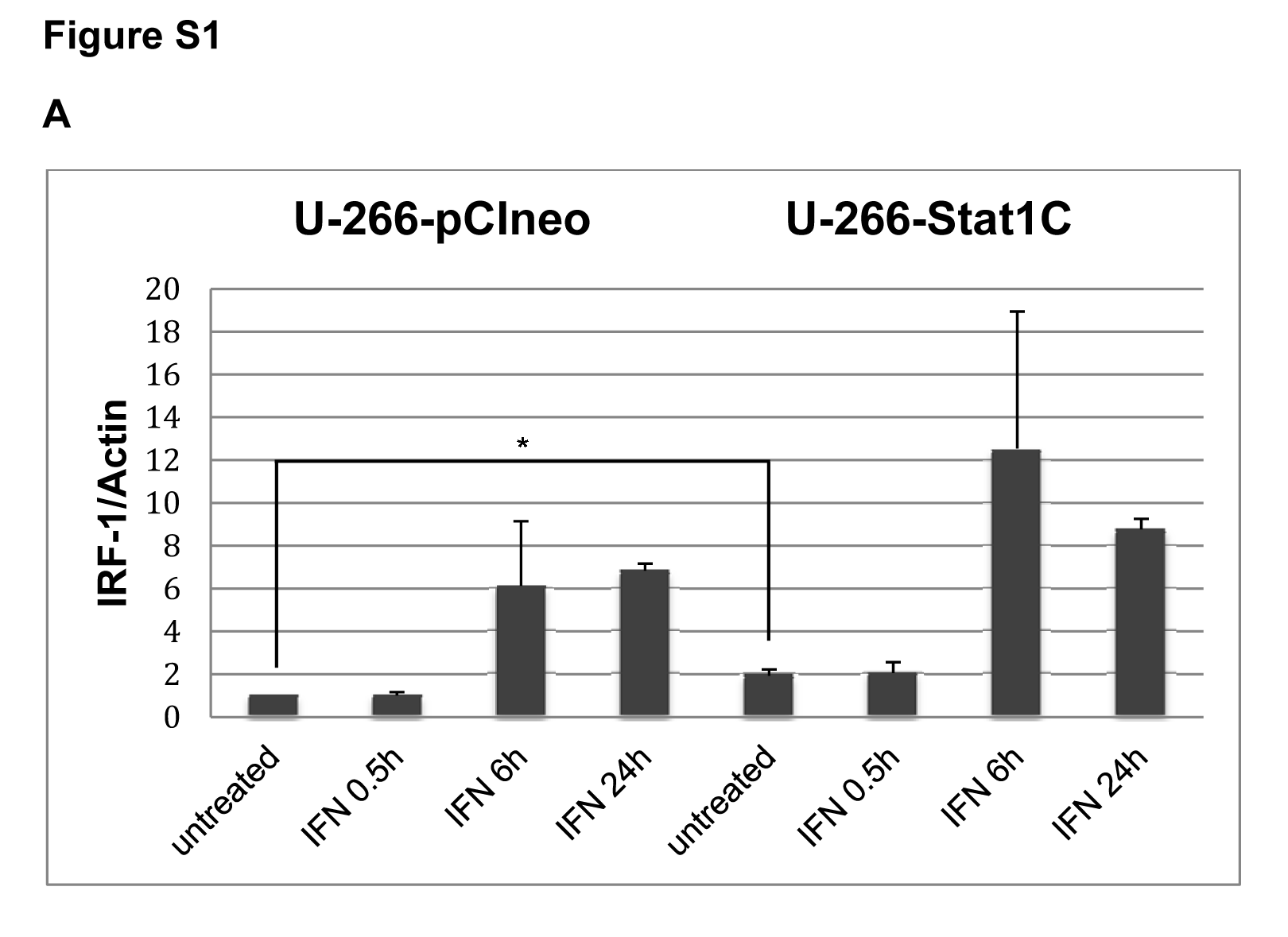
**

**Figure legend S1.** (A) U-266-1970-pCIneo and U-266-1970-Stat1C were induced by IFN-γ (1000U/mL) for the indicated times. Quantification of w.b. for independent biological replicates (n=3). A representative experiment is depicted in Fig 2B. Expression of IRF-1 relative to Actin ±S.D. is shown. * p-value<0.024

**Figure S2**


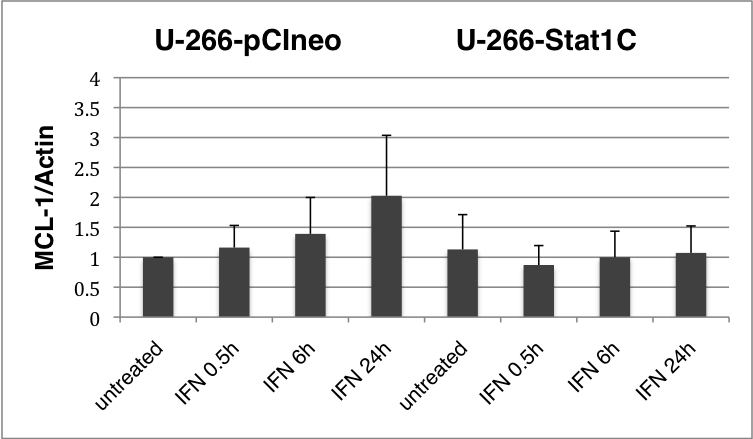


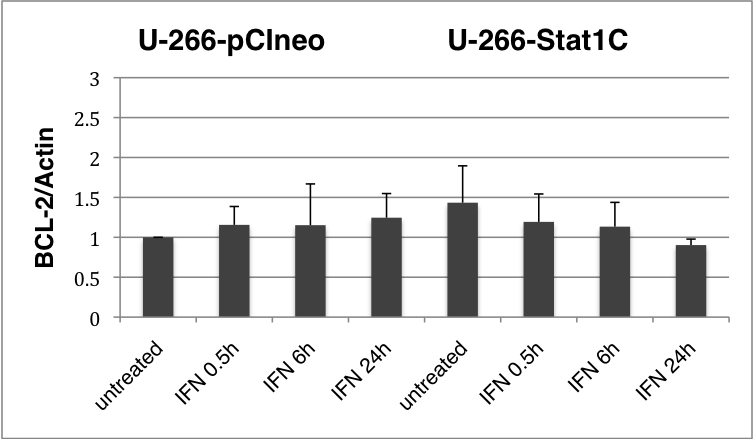


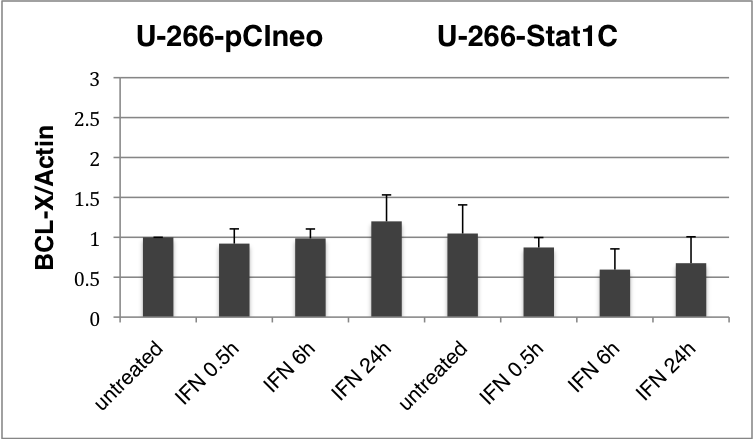


**Figure legend S2.** U-266-1970-pCIneo and U-266-1970-Stat1C were induced by IFN-γ (1000U/mL) for the indicated times. Quantification of w.b. for independent biological replicates (n=3). A representative experiment is depicted in Fig 4B. Expression of MCL-1, BCL-2 and BCL-XL relative to Actin ±S.D. is shown.

**Figure S3**

**A**


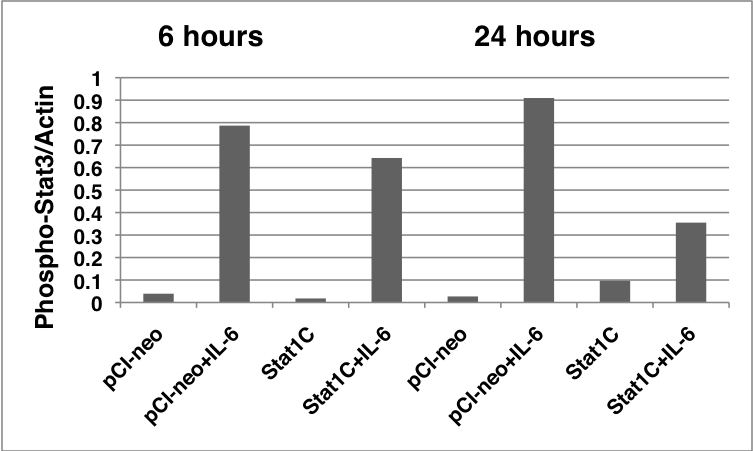


**B**


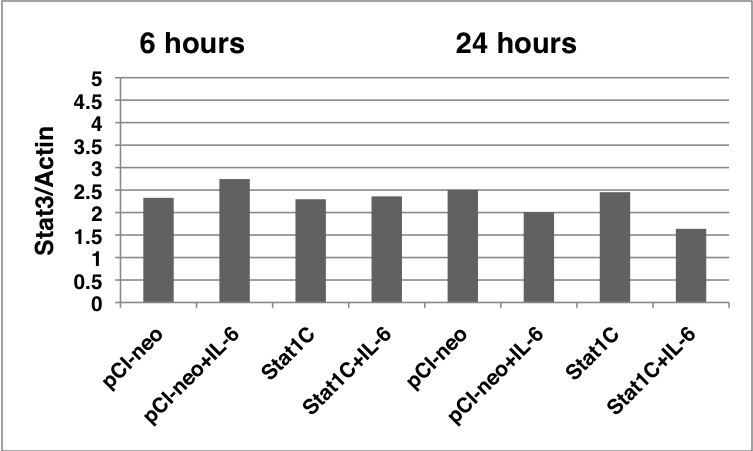


**Figure legend S3.** Quantification of the w.b. shown in Figure 5. (A) phospho-Stat3 expression in U-266-pCIneo and U-266-Stat1C treated with IL-6 (20U/mL) for 6 and 24 hours, and (B) showing total Stat3 expression for the same experiment.

**Figure S4**

**
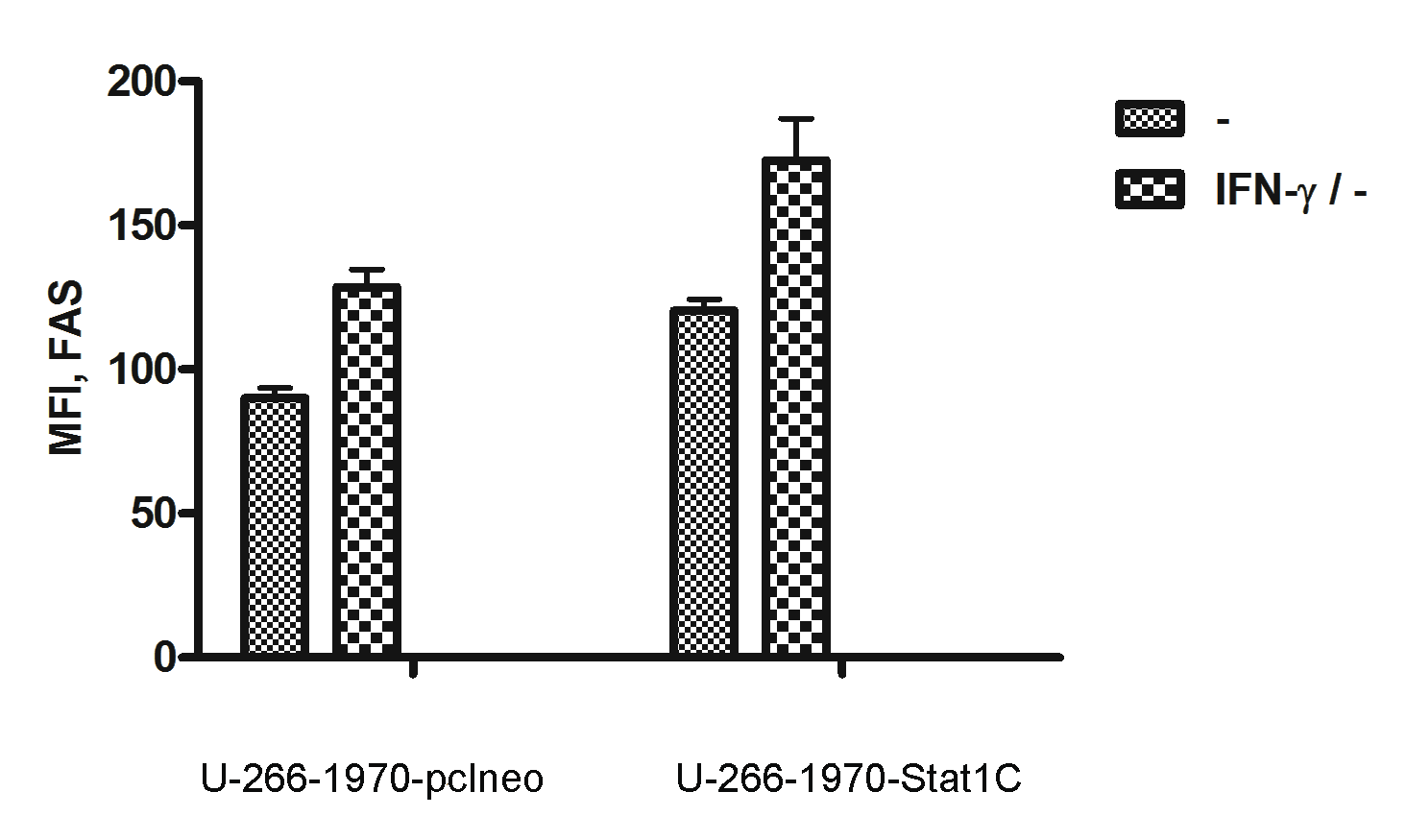
**

**Figure legend S4.** U-266-1970-pCIneo and U-266-1970-Stat1C were induced by IFN-γ (1000U/mL) for 96h. Apoptosis was induced as shown in Fig 6. Expression of CD95/Fas analysed by Flow cytometry analysis (n=3) is indicated as mean flourescense intensity (MFI).

**Figure S5**


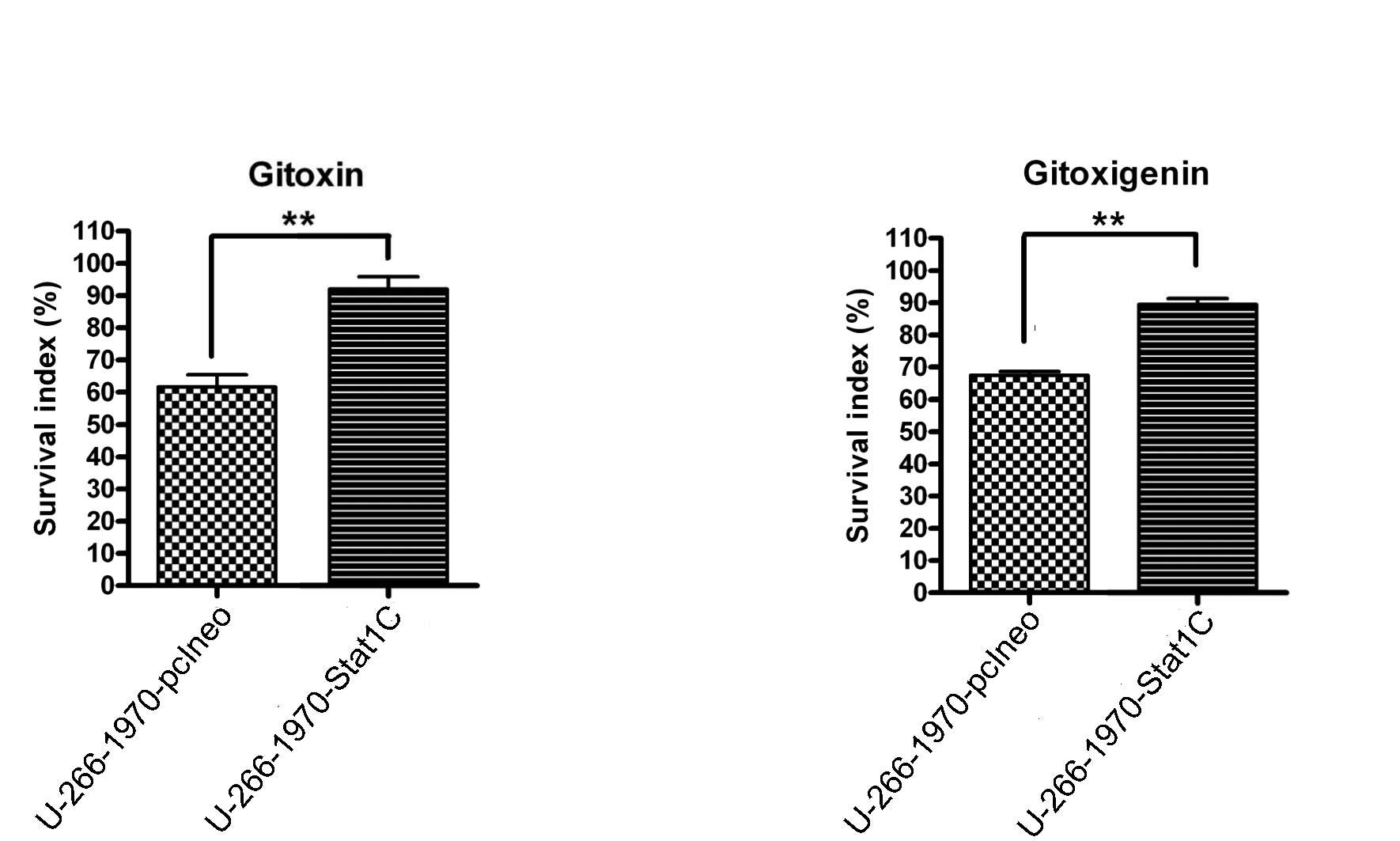


**Figure legend S5.** Response to Gitoxin and Gitoxigenin at 72 h in U-266-1970-pcIneo cells and U-266-1970-Stat1C cells using the Resazurin assay. Each data point represents the mean of three independent experiments ±SD.
